# Assessment of Probiotics Mixture on Memory Function, Inflammation Markers, and Oxidative Stress in an Alzheimer's Disease Model of Rats

**DOI:** 10.29252/ibj.24.4.220

**Published:** 2020-02-29

**Authors:** Shima Mehrabadi, Seyed Shahabeddin Sadr

**Affiliations:** 1 Department of Physiology, School of Medicine, Tehran University of Medical Sciences, Tehran, Iran;; 2 Electrophysiology Research Center, Neuroscience Institute, Tehran University of Medical Sciences, Tehran, Iran

**Keywords:** Alzheimer’s disease, Inflammation, Probiotics, Oxidative stress

## Abstract

**Background:**

The most important cause of neurodegeneration in AD is associated with inflammation and oxidative stress. Probiotics are microorganisms that are believed to be beneficial to human and animals. Probiotics reduce oxidative stress and inflammation in some cases. Therefore, this study determined the effects of probiotics mixture on the biomarkers of oxidative stress and inflammation in an AD model of rats.

**Methods:**

In this study, 50 rats were allocated to five groups, namely control, sham, and AD groups with Aβ1-40 intra-hippocampal injection, as well as AD + rivastigmine and AD + probiotics groups with Aβ1-40 intra-hippocampal injection and 2 ml (10^10^ CFU) of probiotics (*Lactobacillus reuteri*,* Lactobacillus rhamnosus*, and *Bifidobacterium infantis*) orally once a day for 10 weeks. MWM was used to assess memory and learning. To detect Aβ plaque, Congo red staining was used. Oxidative stress was monitored by measuring the MDA level and SOD activity, and to assess inflammation markers (IL-1β and TNF-α) in the hippocampus, ELISA method was employed.

**Results:**

Spatial memory improved significantly in treatment group as measured by MWM. Probiotics administration reduced Aβ plaques in AD rats. MDA decreased and SOD increased in the treatment group. Besides, probiotics reduced IL-1β and TNF-α as inflammation markers in the AD model of rats.

**Conclusion:**

Our data revealed that probiotics are helpful in attenuating inflammation and oxidative stress in AD.

## INTRODUCTION

Alzheimer’s disease is a progressive, neuro-degenerative disease most often characterized by initial cognitive decline and appearance of Aβ plaques in brain. Although the cause of AD is not known, activated microglia and released proinflammatory cytokines have the most important role of neuroinflammation and neurodegeneration in AD^[^^1^^-^^4^^]^. In addition, chronic oxidative stress in AD brains provokes inflammation through the nuclear factor-kappa B signaling pathway, which in turn, accelerates the aging process^[^^5^^,^^6^^]^. 

The gut microbiota is defined as the largest reservoir of microbes (about 10^14^) in the human body^[^^7^^]^. Studies have shown that there is a bidirectional communication between gut and other organs^[^^8^^,^^9^^]^. Disturbances in this cross talks could result in certain diseases like irritable bowel syndrome, inflammatory bowel disease, depression, anxiety, and neurodevelopmental disorders such as autism and Parkinson’s disease, as well as other neurodegenerative and neuroinflammatory disorders like AD^[^^10^^-^^12^^]^. A recent study has suggested that gut microbiota alters in AD patients and may involve in the pathogenesis of AD^[^^13^^]^. In fact, the specific role of gut microbiota is to modulate neuroimmune functions, which indicates that GIT may significantly influence the process of neurodegeneration, especially in association with AD^[^^14^^-^^17^^]^. It has also been reported that gut microbiota is a key player in regulating the innate and adaptive immune response and could affect inflammation responses^[^^8^^,^^9^^]^. 

Probiotics are live microbial food supplements with certain benefits for consumers and are thought to maintain or improve the intestinal microbial balance^[^^18^^]^. Probiotics have been displayed to improve brain-gut-microbiota axis^[^^19^^,^^20^^]^ and regulate nervous system through neuroendocrine, neurometabolic and neuroimmunologic mechanisms^[^^21^^,^^22^^]^. They can also reduce some oxidative stress biomarkers and inflammatory cytokines^[^^23^^]^.

There are few studies on the effect of probiotics on AD and neuroinflammation associated with this disease; however, the underlying mechanism remains still unclear. To this end, this study was undertaken to investigate the impact of probiotics *Lactobacillus reuteri*,* Lactobacillus rhamnosus*, and *Bifidobacterium infantis* on memory function, neuroinflammation, and oxidative stress in an AD model of rats.

## MATERIALS AND METHODS

**Animals**

Fifty male Wistar rats (200-250 g) were used in this study. Each animal was housed in one cage and had free access to food and water^[^^24^^]^. Rats were randomly divided into five groups (n = 10 per group): (1) control group, without any intervention and injection; (2) sham group, with PBS intrahippocampal injection without any dietary plan; (3) Alzheimer group, with Aβ1-40 intrahippocampal injection without any dietary plan; (4) Alzheimer-probiotics (A + P) group, with Aβ1-40 intrahippocampal injection and receiving 2 g (10^10 ^CFU) probiotics (*Lactobacillus reuteri*,* Lactobacillus rhamnosus*, and *Bifidobacterium infantis*) orally once a day for 10 weeks; (5) AD + rivastigmine group, receiving rivastigmine (0.6 mg/kg) orally once a day for two weeks. The dietary plan started five weeks before injecting Aβ1-40 and continued for five weeks after injection. Following behavioral studies, rats were anesthetized with the intraperitoneal injection of ketamine (90 mg/kg) and xylazine (5 mg/kg) to extract brain, which was then stored in -70 °C for ELISA studies and oxidative stress measurement.

**Alzheimer’s model preparation**

**Fig. 1:**
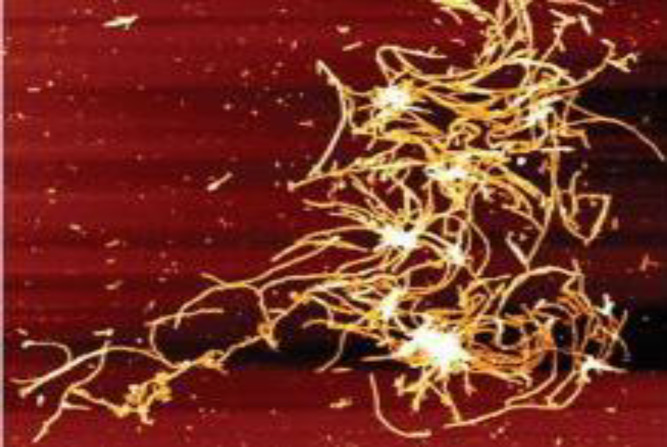
Atomic force microscopy of Aβ1-40 peptides after being dissolved in PBS and incubated for one week. The Figure shows many fibril clusters from Aβ1-40 peptides aggregation. Scan size = 500 × 500 nm

In the first step, to make oligomer Aβ1-40 peptides, 1 mg of Aβ dissolved in 200 μl of PBS was incubated at 37 °C for one week. Then the atomic force microscopy was used to approve oligomer formation (Fig. 1). To induce AD, rats were anesthetized with ketamine (70 mg/kg) + xylazine (10 mg/kg) and then placed in a stereotaxic device. After shaving and puncturing the skull, 2 μl of Aβ1-40 solution was injected into right dorsal hippocampus (CA-1 region) using a Hamilton syringe according to Paxinos and Watson atlas (AP = -4.2, ML = 3, DV = 3.5)^[25]^. Fourteen days after recovery, animals entered the study.

**Probiotics preparation**

Probiotics drop containing *Lactobacillus reuteri*,* Lactobacillus rhamnosus*, and *Bifidobacterium infantis* was obtained from Mahya Darou Company (Tehran, Iran). The probiotics drop (2 ml) was dissolved in 30 ml of tap water; the solution contained 10^10 ^CFU probiotics. Water consumption was monitored. In the treatment group, probiotics consumption continued daily for 10 weeks.

**Behavioral study**

MWM was used to assess the spatial memory of rats after 10 weeks of Aβ injection. A pool with black color and the diameter of 140 cm and depth of 35 cm was used in this study and filled with water (25 ±  2 °C). The pool had four quadrants, one of which was target quadrant with a plexiglass platform placed in it. The platform was invisible and placed 1 cm under water. Rats were trained for four days, and probe test was conducted in one day. Each training day contained four trials that took 60 s. In each trial, rats should find the platform and if they fail to do so, they were placed on the platform to learn its place. Animals were allowed to rest for 15 s, and then the next trial started. 

Finally, in probe test (5^th^ day of the test), the platform was removed from pool, and rats were released randomly to one quadrant and they were allowed to swim for 60 days. The time of rats presence in the target quadrant was calculated^[^^26^^]^. All steps were recorded with a camera above the center of the pool, and the time elapsed, velocity, and the time spent in the target quadrant in the probe test were calculated using Radyab software^[^^4^^]^.

**Aβ** **plaques detection**

To detect Aβ plaques, two rats were chosen randomly, their brains were extracted and kept in 15% formalin for 72 h. Paraffin-sliced sections with 5 µm thick were prepared and stained by Congo red to view amyloid plaques. Then the plaques were observed by an optical microscope with the magnification of 400×^[^^27^^]^.

**MDA** **level measurement**

MDA level was detected by thiobarbituric acid method in brain tissue. Four rats were chosen randomly from any group, and their brains were extracted and homogenized in ice with 0.1 M of phosphate buffer. The brains were then centrifuged at 300 ×g at 4 °C for 10 min, and supernatants were collected. Finally, thiobarbituric acid kit (Nalondi™-Lipid Peroxidation Assay Kit-MDA, Navandsalamat Co., Iran) was used to determine the MDA level in the supernatant.

**SOD enzyme activity** **measurement**

SOD enzyme activity was assessed by colorimetric commercial kit (ZellBio GmbH, Ulm, Germany). SOD activity was determined as the amount of sample catalyzes the decomposition of 1 μmol of superoxide radical into hydrogen peroxide and molecular oxygen in one minute. In this assessment, 65 μL of phosphate buffered saline (pH 7.4) and 30 μL of 3-(4,5-dimethylthiazol- 2-yl)-2,5diphenyltetrazolium bromide (1.25 mM), along with 75 μL of pyrogalum (100 μM), were mixed with 10 μL of homogenized hippocampus tissue and incubated at room temperature for 5 min. Next, 0.75 μL of DMSO was added to the mixture, and the light absorption was read by ELISA atthe wavelength of 420 nm^[^^28^^]^.

**IL-1β and TNF-α levels measurement**

To measure proinflammatory cytokines, four rats were chosen and their brains were extracted and homogenized in ice with 0.1 M of phosphate buffer with protease inhibitor cocktail (1.5 mM of Pepstatin A, 104 mM of 4-benzenesulfonyl fluoride hydrochloride, 80 μM of aprotinin, 4 mM of bestatin, 1.4 mM of E-64, and 2 mM of Leupeptin,) with a ratio of protease inhibitor to sample (1:100). The homogenized brain samples were then centrifuged at 300 ×g at 4 °C for 20 min, and supernatants were collected and used for IL-1β and TNF-α ELISA kits (R&D Systems, USA).

**Statistical analysis**

GraphPad Prism 7.0 was used to analyze the data. All data were shown with mean ± SEM. In behavioral study, two-way ANOVA, followed by post hoc Tukey's test was applied to compare the results of different days (escape latency). In other studies, one-way ANOVA was used to compare the results between groups. *p* < 0.05 was considered statistically significant.

**Ethical statement**

The above-mentioned sampling/treatment protocols were approved by the Ethics Committee for the Care and Use of Laboratory Animals at the Tehran University of Medical Sciences, Tehran, Iran (ethical code: IR.TUMS.MEDICINE.REC.1397.376).

## RESULTS

**Probiotics mixture improved A** **β-** **induced spatial learning, and memory impairment**

**Fig. 2:**
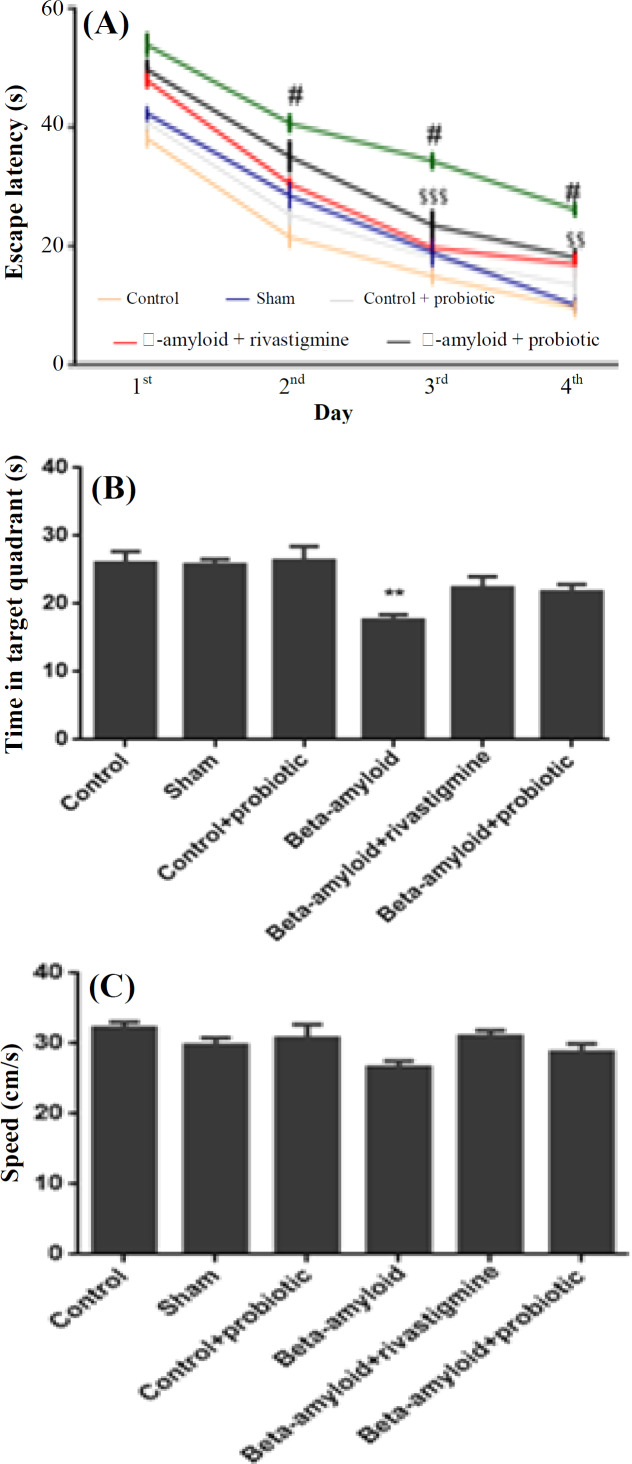
Improvement of Aβ-induced spatial learning and memory impairment by probiotics mixture. (A) Analysis of escape latency (time to find hidden platform) showed a significant difference between Aβ-treated animals and control and sham group (^#^*p* < 0.001). In addition, escape latency data denoted significant difference between Aβ group and Aβ + probiotics group (^$$$^*p* < 0.001 and ^$$^*p* < 0.01). (B) Data from probe test revealed that Aβ-treated animals had significantly less time in the target quadrant in comparison with other groups (^**^p < 0.01). (C) Swimming speed analysis showed no significant difference between the groups (n = 10 rats per group). In 2B and 2C, one-way ANOVA with post-hoc Tukey's test were used to analyze the data. Data were represented as mean ± SEM

To assess spatial memory and learning, MWM was used. Escape latency to arrive to the hidden platform showed that from the first day until the fourth day of training phase, spatial memory improved in all groups. Aβ-treated group had longer time latency in comparison with the control and sham groups in MWM training phase (*p* < 0.001). Administration of probiotics promoted spatial memory and learning in comparison with Aβ-treated group (*p* < 0.01). The probiotics-treated group had no difference with the positive control (rivastigmine + Aβ-treated group), meaning that probiotics had a treatment effect similar to rivastigmine (Fig. 2A). In addition, during the probe test, time was measured in target quadrant that contained hidden platform previously. In Aβ-treated group, time in target quadrant diminished in comparison with the control and sham groups (*p* < 0.01). These data indicated that rats treated with Aβ could not remember that in which quadrant the hidden platform was placed. However, probiotics administration improved their spatial memory, and there was no difference between the probiotics-treated group and the control and sham groups (Fig. 2B). The analysis of swimming speed indicated no significant difference between the groups. These data showed no disability of motor functions in all the groups (Fig. 2C). 

**Probiotics mixture** **diminished Aβ deposition in hippocampus of Aβ-treated rats**

**Fig. 3:**
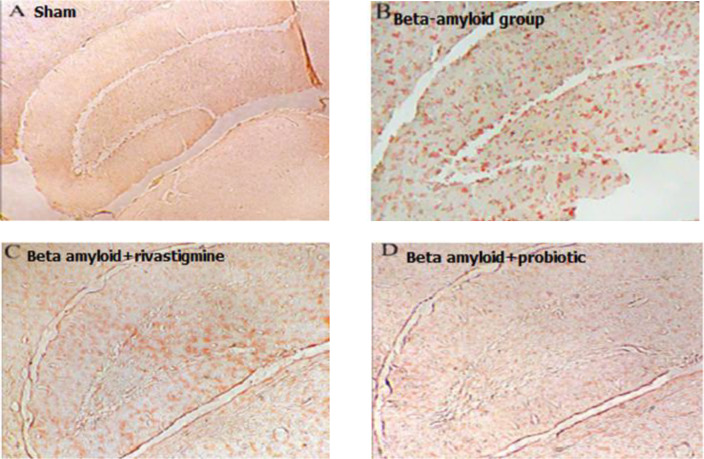
Reduction of Aβ deposition in the hippocampus of Aβ-treated rats by Probiotics mixture. CA-1 region neuronal sections. Administration of Aβ intrahippocampal caused Aβ deposition in hippocampus in comparison with sham group. Ten weeks administration of probiotics had a positive effect on Aβ plaques inhibition even better than Aβ + rivastigmine group (positive control).

To determine Aβ deposition in hippocampus of rats, Congo red staining was used. Based on the Figure 3, Aβ injection in the right hippocampus (CA-1 region) could make Aβ plaques in the brain of rats. Probiotics mixture in animals treated with Aβ could inhibit making Aβ deposition in hippocampus; therefore, in this group, Aβ plaques were disappeared. In addition, the group that received rivastigmine (the positive control), Aβ deposition diminished, but probiotics had a better effect on the clearance of Aβ deposition in rats' brains.

**Probiotics mixture reduced** **MDA** **level and augmented SOD activity in Aβ-treated group**

**Fig. 4:**
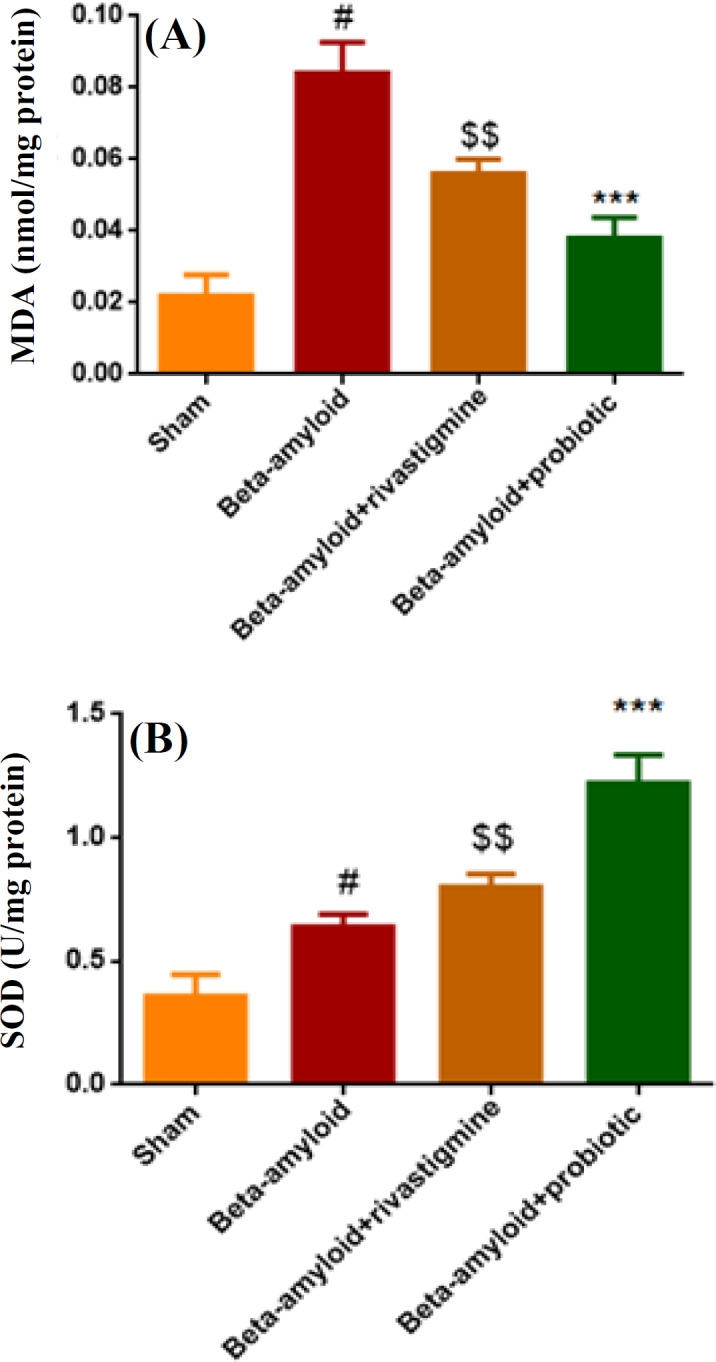
Reduction of MDA level and augmentation of SOD activity in Aβ-treated group by probiotics mixture. (A) MDA level increased significantly in Aβ group in comparison with the sham group (^#^*p* < 0.001); rivastigmine and probiotics reduced MDA activity versus Aβ group (^$$^*p* < 0.01 and ^***^p < 0.001). (B) SOD activity increased significantly in all groups in comparison with sham group (^#^*p* < 0.01, ^$$^*p* < 0.01, ^***^p < 0.001). Data were represented as mean ± SEM (n = 4). One-way ANOVA was used as statistical analysis with post-hoc Tukey's test. ^#^shows the difference between Aβ group vs. sham group; ^$^shows the difference between Aβ group vs. Aβ + rivastigmine; ^*^shows the difference between Aβ group vs. Aβ + probiotic

In this study, to measure oxidative stress level, MDA was used (Fig. 4A). As expected, Aβ injection increased MDA level as an important oxidative marker in comparison with the sham group (*p* < 0.001). However, administration of probiotics mixture demonstrated a significant decrement in MDA level in comparison with Aβ-treated group (*p* < 0.001). Although rivastigmine could decrease MDA level in comparison with Aβ-treated group (*p* < 0.01), probiotics administration had more potent antioxidant activity versus rivastigmine + Aβ-treated group. SOD activity, as an antioxidant enzyme, was measured and increased versus sham group (*p* < 0.05). In addition, in rivastigmine + Aβ-treated group, SOD activity increased in comparison with the sham group (*p* < 0.01) and more than Aβ-treated group (Fig. 4B). Also, probiotics administration could augment SOD activity versus the sham group (*p* < 0.001), and probiotics had a positive effect even more than rivastigmine on increasing SOD activity.

**Probiotics mixture reduced inflammation markers level (IL-1β and TNF-α) in Aβ-treated group**

**Fig. 5:**
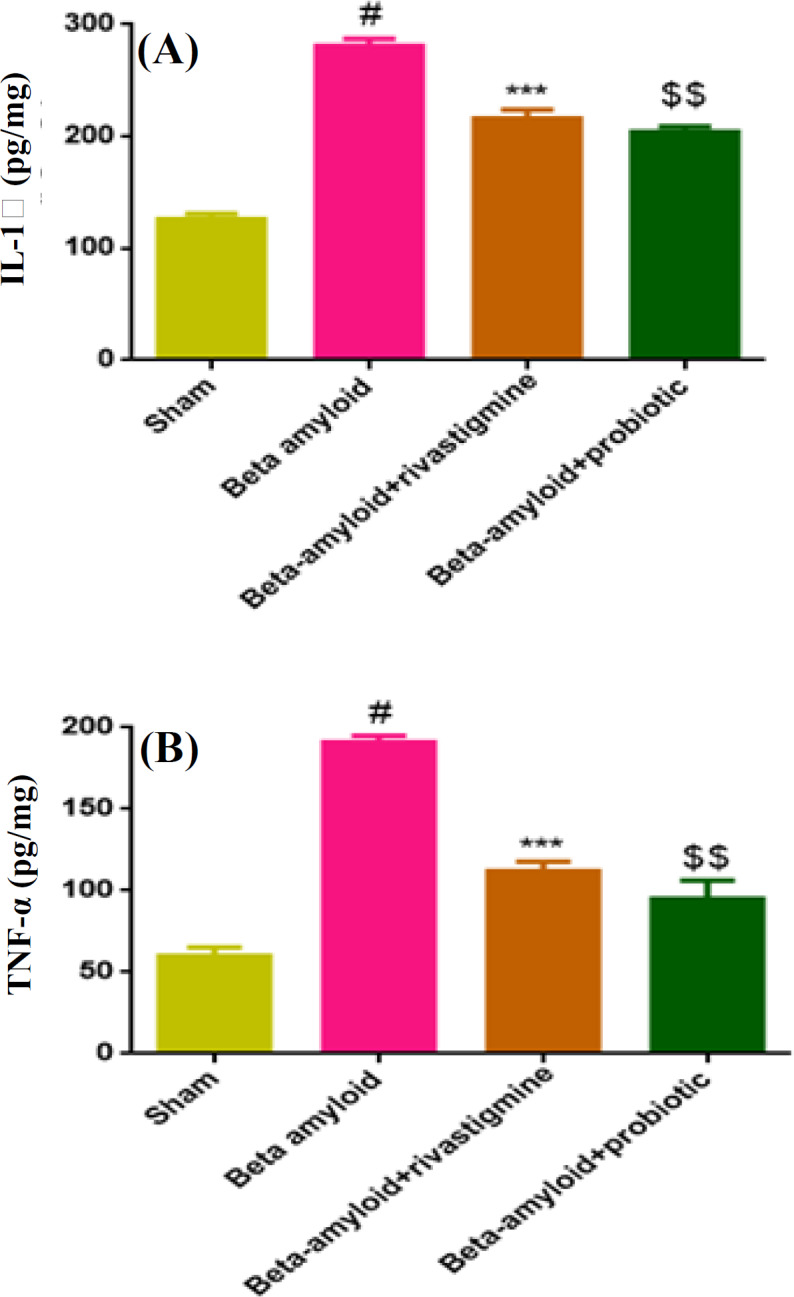
Reduction of inflammation makers level (IL-1β and TNF-α) in Aβ-treated group by probiotics mixture. Data were represented as mean ± SEM (n = 4). In Aβ-treated group, IL-1β (A) and TNF-α (B) increased versus sham group (^#^*p *< 0.01). Treatment with probiotics mixture in Aβ-treated rats decreased IL-1β and TNF-α levels like rivastigmine group (^***^p < 0.001, ^$$^*p* < 0.001). One-way ANOVA was used as statistical analysis with post-hoc Tukey’s test. ^#^ shows the difference between Aβ group vs. sham group; ^*^ shows the difference between Aβ group vs. Aβ + rivastigmine; ^$^ shows the difference between Aβ group vs Aβ + probiotics

Aβ-treated rats significantly exhibited an elevation of IL-1β (*p* < 0.01) and TNF-α (*p* < 0.01) levels in hippocampal tissue supernatant compared to the sham group (Fig. 5). Probiotics mixture could also decrease IL-1β (*p* < 0.01) and TNF-α (*p* < 0.01) levels significantly in comparison with Aβ-treated rats. As data showed, probiotics mixture attenuated inflammation markers in hippocampus tissue in comparison with Aβ intrahippocampal microinjection group.

## DISCUSSION

The present study revealed that probiotics play an effective role in the improvement of memory impairment in the AD model of rats, as well as of memory and learning in the MWM test. In our histological study, probiotics administration decreased Aβ aggregation in hippocampus in an AD model. In addition, probiotics attenuated neuroinflammation by reducing inflammation and oxidative stress markers in animals with Aβ intrahippocampal microinjection. Evidence has indicated that oxidative stress acts as a trigger for the deposition and accumulation of Aβ in AD through the production of reactive oxygen species^[^^29^^-^^31^^]^. Our data showed that the administration of probiotics mixture for 10 weeks improved delay in escape latency in the AD model of rats in MWM test. This finding suggested a significant improvement in spatial memory consolidation. Our finding also showed that probiotics reduced augmented MDA level, as an oxidative marker, and IL-1β and TNF-α levels, as important inflammation markers. 

SODs convert superoxide to hydrogen peroxide, which is removed by glutathione peroxidase or catalase^[^^32^^,^^33^^]^. In the present study, probiotics increased the SOD enzyme level, indicating that probiotics have an antioxidative effect on the AD model. It has also been shown that probiotics play the same role in GIT and reduce intestinal oxidative stress and neuroinflammatory cytokines in several experimental models^[^^34^^-^^36^^]^. There are several theories on the relationship between alternation in microbiota and neuroinflammatory disorders. Gut microbiota is required for normal immune system maturation, which can influence the adaptive and innate immune systems in completely different ways^[^^37^^-^^39^^]^. 

Studies on MS, as a neuroinflammation disorder, have shown that probiotics can be helpful in reducing inflammatory cytokines in MS patients. Probiotics supplement decreased IL-6 levels and increased IL-10 concentration (as an anti-inflammatory cytokine) in the serum of MS patients^[^^40^^]^. Probiotics treatment improved clinical symptoms by creating a balance in inflammatory and anti-inflammatory responses in MS patients^[^^9^^,^^40^^]^. In fact, probiotics mixture can be useful in the treatment of neurodegenerative and neuro-inflammatory diseases through their immune-modulatory effects^[^^41^^,^^42^^]^. There are many relationships between gut and brain at molecular levels. The gut–brain axis is a dynamic bidirectional neuroendocrine system describing the connections between the GIT and the nervous system^[^^43^^,^^44^^]^. There are many common regulatory factors between the enteric nervous system and the CNS. Studies have revealed that gut microbiota dysbiosis is related to many neuroinflammatory and neurodegenerative diseases^[44]^. Microbiota-associated molecular patterns can activate the host innate immune system via pattern-recognition receptors such as toll-like receptors and nucleotide-binding domain and leucine-rich repeat containing receptors (NOD-like receptors) that are present in intestinal epithelial and myeloid cells^[^^42^^]^. Thus, the activation of toll-like receptors and NOD-like receptors could be implicated in the mechanisms by which gut microbiota trigger many neuroimmune disorders^[^^45^^]^. The beneficial effects of probiotics therapy is likely the improvement of the intestinal barrier function, which leads to the prevention of a continuous stimulation of the host innate immune system by the gut microbiome and inhibition of releasing proinflammatory factors in blood flow, in order to advance neuroinflammation in CNS^[^^46^^]^. In general, there is a loss of gut microbial diversity in the aging gut. In a few studies, there were significant variations in the composition of the gut microbiota in aging patients suffering from neurodegenerative diseases^[^^47^^,^^48^^]^. Various probiotics produce different neuroactive molecules that directly or indirectly impact signaling in the CNS. There are many interlocking hormonal and biochemical pathways relating GIT health to the brain and creating a strong therapeutic potential for probiotics use against neuro-degeneration^[^^42^^]^. In previous studies, probiotics mixture of* Lactobacillus reuteri*,* Lactobacillus rhamnosus*, and *Bifidobacterium infantis* succeeded in the treatment of many inflammatory bowel diseases^[^^49^^-^^51^^]^. Our study also revealed that probiotics mixture of the three mentioned bacteria can slow down the progression of oxidative stress and neuroinflammation in AD model of rats.

The findings of the present study demonstrated that the 10-week consumption of probiotics in the AD model of rats had favorable effects on memory deficit, oxidative stress markers, and proinflammatory cytokines. This study clarified that probiotics can serve as an effective treatment in many neurodegenerative diseases along with other effective drugs in AD patients.
